# Association of patient characteristics with clinical outcomes in a cohort of hospitalised patients with SARS-CoV-2 infection in a Greek referral centre for COVID-19

**DOI:** 10.1017/S0950268822001364

**Published:** 2022-08-16

**Authors:** I. Rapti, A. Asimakopoulos, A. Liontos, M. Kosmidou, E. Christaki, D. Biros, O. Milionis, S. Tsourlos, E. Ntotsikas, E. Ntzani, E. Evangelou, K. Gartzonika, I. Georgiou, I. Tzoulaki, K. Tsilidis, H. Milionis

**Affiliations:** 1 Department of Internal Medicine, Ioannina University Hospital, University of Ioannina, Ioannina, Greece; 2 Clinical and Molecular Epidemiology Unit, Department of Hygiene and Epidemiology, School of Medicine, University of Ioannina, Ioannina, Greece; 3 Center for Evidence-Based Medicine, Department of Health Services, Policy and Practice, School of Public Health, Brown University, Providence, RI, USA; 4 Department of Epidemiology and Biostatistics, School of Public Health, Imperial College London, London, UK; 5 Microbiology Department, Faculty of Medicine, School of Health Sciences, University of Ioannina, Ioannina 45110, Greece; 6 Genetics and IVF Unit, Department of Obstetrics and Gynaecology, Medical School, University of Ioannina, Ioannina 45110, Greece

**Keywords:** COVID-19, hospitalisation, intubation, mortality, risk factors

## Abstract

Patient-important outcomes related to coronavirus disease 2019 (COVID-19) continue to drive the pandemic response across the globe. Various prognostic factors for COVID-19 severity have emerged and their replication across different clinical settings providing health services is ongoing. We aimed to describe the clinical characteristics and their association with outcomes in patients hospitalised with COVID-19 in the University Hospital of Ioannina. We assessed a cohort of 681 consecutively hospitalised patients with COVID-19 from January 2020 to December 2021. Demographic data, underlying comorbidities, clinical presentation, biochemical markers, radiologic findings, COVID-19 treatment and outcome data were collected at the first day of hospitalisation and up to 90 days. Multivariable Cox regression analyses were performed to investigate the associations between clinical characteristics (hazard ratios (HRs) per standard deviation (s.d.)) with intubation and/or mortality status. The participants' mean age was 62.8 (s.d., 16.9) years and 57% were males. The most common comorbidities were hypertension (45%), cardiovascular disease (19%) and diabetes mellitus (21%). Patients usually presented with fever (81%), cough (50%) and dyspnoea (27%), while lymphopenia and increased inflammatory markers were the most common laboratory abnormalities. Overall, 55 patients (8%) were intubated, and 86 patients (13%) died. There were statistically significant positive associations between intubation or death with age (HR: 2.59; 95% CI 1.52–4.40), lactate dehydrogenase (HR: 1.44; 95% CI 1.04–1.98), pO_2_/FiO_2_ ratio < 100 mmHg (HR: 3.52; 95% CI 1.14–10.84), and inverse association with absolute lymphocyte count (HR: 0.54; 95% CI 0.33–0.87). These data might help to identify points for improvement in the management of COVID-19 patients.

## Introduction

The coronavirus disease 2019 (COVID-19) pandemic has been associated with an increasing burden of hospitalisations due to pneumonia and multiorgan disease. COVID-19 is caused by the severe acute respiratory syndrome coronavirus 2 (SARS-CoV-2). The clinical presentation ranges from asymptomatic up to a wide spectrum of symptoms, including mild symptoms of upper respiratory tract infection or life-threatening sepsis [1]. Since December 2019 and up to June 2022, COVID-19 has resulted in an estimated number of 532 201 219 infections, and 6 305 358 estimated total deaths [2]. Recent reports suggest that approximately 33% of hospitalised patients developed acute respiratory distress syndrome (ARDS) at a median time of 8 days from symptom onset [3]. In these reports [3, 4], critically ill patients were older, mainly male with underlying comorbidities. The case fatality rates for COVID-19 range from 0.27% to 10%, possibly explained by differences in demography, testing strategies and prevalence of underlying conditions [5].

Limited information has been available to describe the presenting characteristics and outcomes of Greek patients with COVID-19 requiring hospitalisation. The objective of this study is to describe the clinical characteristics, outcomes and the emerging associations thereof for 681 consecutive patients hospitalised with COVID-19 at the University Hospital of Ioannina between January 2020 to December 2021.

## Methods

### Study participants

The study site was the University Hospital of Ioannina. The Ioannina COVID-19 cohort comprises 681 patients meeting the following criteria: (1) patients with SARS-CoV-2 infection confirmed by positive polymerase chain reaction (PCR) testing of nasopharyngeal specimens, (2) hospitalisation and (3) age 18 years or older. Medical records for all patients with SARS-CoV-2 detected in nasopharyngeal swabs between January 2020 and December 2021 were reviewed. All outcomes were monitored for 90 days.

### Data collection and definitions

Data collection was performed by a team of physicians and research associates, who retrospectively reviewed the health records to obtain data on a standardised data collection form. For patients who met eligibility criteria, the following groups of variables were collected from the medical records: demographic data, anthropometric parameters, underlying comorbidities, vaccination status, symptoms and signs at presentation, clinical course during hospitalisation, disease severity, laboratory parameters, radiologic findings, administered treatments, complications and outcomes and time thereof. All variables were recorded for the day of admission and then consecutively up to the 90th day once data were available.

Demographic parameters included age and sex. Anthropometric parameters included weight, height and body mass index (BMI) calculated as weight in kilograms divided by height in metres squared [6]. Each patient self-reported the vaccination status as unvaccinated, partially or fully vaccinated. Vital signs included systolic and diastolic blood pressure, heart rate, temperature (in Celsius scale) and oxygen saturation at admission.

Comorbidities were recorded based on published work on COVID-19 and included: hypertension (defined as systolic blood pressure > 130 mmHg and/or diastolic blood pressure > 80 mmHg [7]), cardiovascular disease (CVD), dyslipidemia (defined as elevated total or low-density lipoprotein (LDL) cholesterol levels or low levels of high-density lipoprotein (HDL) cholesterol [8]), diabetes mellitus, chronic obstructive pulmonary disease (COPD), chronic kidney disease (CKD), obesity (defined as BMI ≥ 30 kg/m^2^), immunosuppression (defined as any condition leading to loss or qualitative deficiency in cellular or humoral immune components including HIV infection, hematopoietic malignancies, treatment with radiation, treatment with immunosuppressive drugs or prolonged treatment with corticosteroids), smoking and other comorbidities.

All symptoms reported at presentation were documented including fever, cough, headache, fatigue, dyspnoea, myalgias, loss of appetite, new loss of taste or smell, nausea, vomiting or diarrhoea, sore throat and rhinorrhea.

Severity of respiratory failure is estimated with the Horowitz Index for Lung Function (pO_2_/FiO_2_ ratio). The Horowitz Index for Lung Function (pO_2_/FiO_2_ ratio) is categorised in four categories: (1) pO_2_/FiO_2_ ratio > 300 mmHg, (2) 200 mmHg < pO_2_/FiO_2_ ratio < 300 mmHg, (3) 100 mmHg < pO_2_/FiO_2_ ratio < 200 mmHg and (4) pO_2_/FiO_2_ ratio < 100 mmHg with values of pO_2_/FiO_2_ ratio lower than 300 mmHg indicating ARDS [9].

Laboratory parameters collected included complete blood cell counts with differential, basic metabolic panel, kidney function, hepatic function, lactate dehydrogenase, creatine phosphokinase, high sensitivity troponin, D-dimers and markers of inflammation (C-reactive protein (CRP), ferritin, interleukin 6 (IL-6)) for all patients.

Radiologic exams included a chest radiograph at baseline for all patients and chest CT scan for most patients. Radiologic exams were repeated once determined clinically relevant by health care practitioners on case-by-case basis. Radiology results were reviewed for the presence of unilateral or bilateral infiltrates, ground glass opacities, effusions, pneumothorax, vascular abnormalities as well as the extent of pulmonary disease.

Treatment modalities according to international and national COVID-19 treatment guidelines [10] included corticosteroids, remdesivir and tocilizumab in addition to supportive measures and management of associated comorbidities and conditions complicating COVID-19 (e.g. thrombosis events).

In the light of accumulating knowledge in the management of COVID-19 patients, primary clinical outcomes were defined as: (1) a composite outcome including respiratory failure and/or ARDS requiring intubation and intensive care unit (ICU) admission or death and (2) death related to COVID-19.

### Statistical analysis

Descriptive statistics were calculated to describe the patients' characteristics and their distribution by mortality status. Based on data type and normality of distribution, continuous variables were described as median (interquartile range (IQR)) or mean (s.d.); categorical variables were described as frequency rates and percentages. Cox proportional hazards regression models adjusted for age and sex were performed to estimate the association of demographic characteristics, comorbidities, symptoms, vital signs, laboratory values, pO_2_/FiO_2_ ratio, radiologic findings, the treatment used, vaccination status with intubation and ICU admission or death (composite outcome) and mortality alone. Continuous variables were standardised so that hazard ratios were comparable per standard deviation increase. The proportionality assumption was investigated using Schoenfeld residuals and was met in all models. The False Discovery Rate (FDR) correction following the Benjamini–Hochberg approach [11] was applied to reduce the probability of false positive findings. Variables with a *P*-value < 0.011 for the composite outcome and *P*-value < 0.015 for mortality were fitted in a multivariable Cox regression model. Hazard ratios (HRs) with 95% confidence intervals (CIs) were reported for all models. Additionally, we checked for collinearity with a variance inflation factor (VIF) < 5 indicating the absence of collinearity between variables included in the multivariable models. Missing data were imputed with values up to the third day of hospitalisation as proxies of baseline variables in case the percentage of missingness was greater than 15% (e.g. ferritin, D-Dimers, interleukin-6 and CT chest). Results were considered statistically significant in the multivariate model if the *P*-value was <0.05. All analyses were performed using Stata (version 13.1; StataCorp, College Station, TX, USA).

## Results

A total of 681 consecutively hospitalised patients with SARS-CoV-2 infection between January 2020 and December 2021 were included. The baseline clinical characteristics of the overall cohort are summarised in Table 1. Male patients comprised 57% (*n* = 389) of the total population. The mean age was 62.8 (s.d.: 16.89) years. The mean BMI was 29.4 (s.d.: 6.05). Most patients (*n* = 600, 88%) reported at least one comorbidity, including hypertension (*n* = 309, 45%), dyslipidemia (*n* = 230, 34%), diabetes mellitus (*n* = 144, 21%) and CVD (*n* = 129, 19%). Most of the hospitalised patients (*n* = 620, 95%) were unvaccinated.

**Table 1.** Baseline characteristics of patients hospitalised with COVID-19 at the University Hospital of Ioannina, Greece from January 2020 to December 2021

Characteristics	Total (*N* = 681)	Alive (*n* = 595)	Deceased (*n* = 86)
Demographic characteristics			
Age, years, mean (s.d.)	62.8 (16.89)	60.8 (16.49)	77.3 (11.91)
Female, *n* (%)	292 (42.88)	248 (41.68)	44 (51.16)
BMI, kg/m^2^, mean (s.d.)^a^	29.4 (6.05)	29.51 (5.22)	27.53 (5.86)
Vaccination status, *n* (%)			
Unvaccinated	620 (95.38)	549 (95.81)	71 (92.21)
Partially vaccinated	12 (1.85)	9 (1.57)	3 (3.90)
Fully vaccinated	18 (2.77)	15 (2.62)	3 (3.90)
Comorbidities			
Hypertension, *n* (%)	309 (45.37)	257 (43.19)	52 (60.47)
CVD, *n* (%)	129 (18.94)	96 (16.30)	33 (38.37)
Diabetes mellitus, *n* (%)	144 (21.18)	119 (20.03)	25 (29.07)
Dyslipidemia, *n* (%)	230 (33.82)	193 (32.49)	37 (43.02)
COPD, *n* (%)	35 (5.14)	24 (4.03)	11 (12.79)
Cancer, *n* (%)	32 (4.70)	22 (3.74)	10 (11.63)
Immunosuppression, *n* (%)	42 (6.19)	31 (5.22)	11 (12.94)
Obesity, *n* (%)	194 (31.14)	174 (32.04)	20 (25)
CKD, *n* (%)	33 (4.85)	21 (3.53)	12 (13.95)
Smoking, *n* (%)	83 (12.41)	75 (12.82)	8 (9.52)
Symptoms			
Fever, *n* (%)	564 (81.06)	502 (84.65)	62 (72.09)
Cough, *n* (%)	337 (49.85)	301 (51.02)	36 (41.86)
Dyspnoea, *n* (%)	181 (26.66)	148 (24.96)	33 (38.37)
Headache, *n* (%)	54 (7.95)	48 (8.09)	6 (6.98)
Fatigue, *n* (%)	192 (28.28)	165 (27.82)	27 (31.40)
Myalgias, *n* (%)	109 (16.05)	99 (16.69)	10 (11.63)
Loss of appetite, *n* (%)	52 (7.67)	39 (6.59)	13 (15.12)
Anosmia, *n* (%)	53 (7.81)	48 (8.09)	5 (5.81)
Ageusia, *n* (%)	60 (8.84)	56 (9.44)	4 (4.65)
Nausea, diarrhoea, vomiting, *n* (%)	216 (31.81)	196 (33.05)	20 (23.26)
Sore throat, *n* (%)	43 (6.33)	38 (6.41)	5 (5.81)
Rhinorrhea, *n* (%)	13 (1.91)	11 (1.85)	2 (2.33)
Vital signs			
Systolic BP, mmHg, mean (s.d.)	133.68 (19.51)	133.77 (19.43)	133.16 (20.19)
Diastolic BP, mmHg, mean (s.d.)	77.43 (12.69)	77.79 (12.46)	74.88 (13.97)
Heart rate, beats/min, mean (s.d.)	83.01 (13.69)	83.02 (13.61)	82.92 (14.31)
SatO_2_%, mean (s.d.)	94.18 (4.05)	94.35 (3.84)	92.94 (5.21)
Temperature, mean (s.d.)	37.09 (0.99)	37.11 (0.99)	36.93 (0.97)
PO_2_/FiO_2_ ratio, *n* (%)			
>300 mmHg	252 (48.93)	233 (52.13)	19 (27.94)
>200 mmHg & ≤300 mmHg	120 (23.30)	105 (23.49)	15 (22.06)
>100 mmHg & ≤200 mmHg	103 (20.00)	85 (19.02)	18 (26.47)
≤100 mmHg	40 (7.77)	24 (5.37)	16 (23.53)
Laboratory values			
White blood cell count, cells/μl, median (IQR)	5755 (4392.5–8057.5)	5685 (4350–7780)	6635 (5137.5–10312.5)
Absolute lymphocyte count, cells/μl, median (IQR)	975 (700–1360)	990 (730–1385)	805 (552.25–1202.5)
Aspartate aminotransferase, IU/l, median (IQR)	36 (26–50)	35 (26–49.25)	39.5 (30–54)
Lactate dehydrogenase, IU/l, median (IQR)	297 (247–406)	290 (245.5–395.5)	390 (268.25–498.5)
Ferritin, ng/ml, median (IQR)^b^	348 (158–637)	336 (149–620.25)	391 (261–743)
D-dimer, μg/ml, median (IQR)^c^	0.76 (0.49–1.36)	0.72 (0.46–1.25)	1.33 (0.81–2.07)
C-reactive protein (IQR), mg/l, median (range)	49 (16–100)	42 (15–95.75)	74 (32–121.25)
Il-6, IU/ml, median (IQR)^d^	22.45 (10.73–43.4)	20.9 (9.75–37.3)	48 (22.15–121.5)
Radiologic Findings (CT scan)^e^			
Normal, *n* (%)	36 (9.86)	34 (10.21)	2 (6.25)
Unilateral infiltrate, *n* (%)	20 (5.48)	16 (4.80)	4 (12.50)
Bilateral infiltrate, *n* (%)	28 (7.67)	22 (6.61)	6 (18.75)
Ground glass opacities, *n* (%)	281 (76.99)	261 (78.38)	20 (62.50)
Vascular tree-in-bud, *n* (%)^f^	104 (30.86)	90 (29.90)	14 (38.89)
Treatment			
Corticosteroids, *n* (%)	512 (76.42)	438 (74.62)	74 (89.16)
Remdesivir, *n* (%)	327 (54.41)	292 (55.62)	35 (46.05)
Tocilizumab,*n* (%)	97 (14.610	69 (11.88)	28 (33.73)
Length of hospitalisation, median (IQR)	11 (7–17)	10 (7–16)	18 (8.25–27)
Complications			
Intubation, *n* (%)	55 (8.08)	17 (2.86)	38 (44.19)

BP, blood pressure; CKD, chronic kidney disease; COPD, chronic obstructive pulmonary disease; CT, computer tomography; CVD, coronary artery disease; Il-6, Interleukin-6; SatO_2_%, oxygen saturation; s.d., standard deviation; IQR, interquartile range (25th–75th quantile).

a *N* = 293, Alive: *n* = 275, Deceased: *n* = 18.

b *N* = 465, Alive: *n* = 408, Deceased: *n* = 57.

c *N* = 555, Alive: *n* = 490, Deceased: *n* = 65.

d *N* = 356, Alive: *n* = 308, Deceased: *n* = 44.

e *N* = 365, Alive: *n* = 333, Deceased: *n* = 32.

f *N* = 337, Male: *n* = 194, Female: *n* = 143.

### Clinical presentation, laboratory markers and radiologic findings at admission

Common symptoms at presentation were fever (*n* = 564, 83%), cough (*n* = 337, 50%), dyspnoea (*n* = 181, 27%), while gastrointestinal symptoms were present in 216 patients (32%). Median duration of symptoms prior to hospitalisation was 7 days (IQR: 3–10).

The mean (s.d.) systolic and diastolic blood pressure were 133.68 (19.51) and 77.43 (12.69) respectively. The mean (s.d.) heart rate and temperature were 83.01 (13.69) and 37.09 °C (0.99) respectively, while the oxygen saturation had a mean (s.d.) of 94.09 (4.05).

Regarding pO_2_/FiO_2_ ratio, 120 patients (23%) presented with mild (200–300 mmHg), 103 patients (20%) with moderate (100–200 mmHg) and 40 patients (8%) with severe ARDS.

At admission, the white blood cell counts had a median (IQR) of 5755/μl (4392.5–8057.5), and the median (IQR) absolute lymphocyte count was 975/μl (700–1360). Median (IQR) values of aspartic transferase (AST) and lactate dehydrogenase (LDH) were 36 IU/l (26–50) and 297 IU/l (247–406), respectively. Inflammatory markers including CRP, and ferritin had median (IQR) values of 49 mg/l (16–100) and 348 ng/ml (158–637), respectively. Intereleukin-6 had a median (IQR) 22.45 IU/ml (10.73–43.40). Median (IQR) value of D-dimers was 0.76 μg/ml (0.87).

A chest CT scan was available for 365 patients at baseline. Findings included unilateral infiltrates (*n* = 21%), bilateral infiltrates (*n* = 29%) and ground glass opacities (*n* = 281, 77%). Vascular tree-in-bud was present in 104 patients (31%).

All clinical symptoms, vital signs, laboratory markers and radiologic findings at admission are summarised in Table 1.

### Administrated therapies during hospitalisation

Therapeutic regimens included corticosteroids (*n* = 512, 76%), remdesivir (*n* = 327, 54%) and tocilizumab (*n* = 97, 15%). Administrated regimens are summarised in Table 1.

### Length of hospitalisation, need for intubation, mortality rate

Mean length of hospitalisation was 11 days (IQR: 7–17). Overall, 103 (15%) patients were either intubated or deceased. In total, 55 patients (8%) required intubation and ICU admission while we observed 86 deaths (13%).

### Associations with respiratory failure and/or acute respiratory distress syndrome (ARDS) requiring intubation and ICU admission or death (composite outcome)

**Fig. 1.**
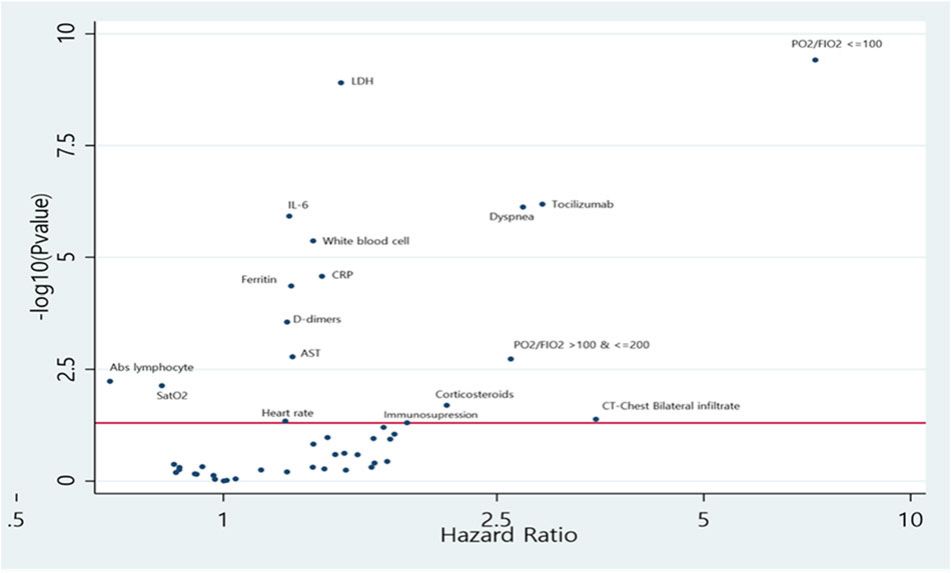
Volcano plot with Hazard ratios and −log10 (*P*-value) of each estimate from Cox regression analysis adjusted for age and sex for composite outcome (intubation and ICU admission or death). *The statistical threshold is 0.05 (red line in *y*-axis). The highest in *y*-axis the lowest *P*-value for the variables.

The Cox regression model adjusted for age and sex revealed a statistically significant inverse association between the composite outcome (intubation and ICU admission or death) and oxygen saturation (HR 0.81; 95% CI 0.70–0.95), absolute lymphocyte count (HR 0.68; 95% CI 0.52–0.90), and positive associations for white blood cells count (HR: 1.35; 95% CI 1.19–1.54), aspartate aminotransferase (HR: 1.26; 95% CI 1.09–1.46), lactate dehydrogenase (HR: 1.48; 95% CI 1.31–1.68), ferritin (HR: 1.26; 95% CI 1.13–1.40), D-Dimers (HR: 1.24; 95% CI 1.10–1.39), CRP (HR: 1.39; 95% CI 1.19–1.62), IL-6 (HR: 1.25; 95% CI 1.14–1.36), presence of immunosuppression (HR: 1.85; 95% CI 1.00–3.42), dyspnoea (HR: 2.73; 95% CI 1.83–4.06), administration of tocilizumab (HR: 2.91; 95% CI 1.91–4.44), use of corticosteroids (HR: 2.11; 95% CI 1.12–3.97), presence of bilateral infiltrates in CT scan (HR: 3.48; 95% CI 11.55), pO_2_/FiO_2_ ratio of 100–200 mmHg (HR: 2.62; 95% CI 1.43–4.80) and pO_2_/FiO_2_ ratio < 100 mmHg (HR: 7.27; 95% CI 3.90–13.52). Absolute lymphocyte count, white blood cells count, aspartate aminotransferase, lactate dehydrogenase, ferritin, D-Dimers, CRP, IL-6, dyspnoea, administration of tocilizumab, pO_2_/FiO_2_ ratio satisfied the FDR correction and were fitted in the multivariate model. Oxygen saturation and pO_2_/FiO_2_ ratio were statistically (*r* = 0.49 in our sample) and clinically correlated, so oxygen saturation was omitted from the multivariable model. All results are summarised on Supplementary Table S1 and presented on a volcano plot on Figure 1.

In the multivariable Cox regression model, the composite outcome of intubation and ICU admission or death was positively associated with age (HR: 2.59; 95% CI 1.52–4.40), lactate dehydrogenase (HR: 1.44; 95% CI 1.04–1.98), pO_2_/FiO_2_ ratio < 100 mmHg (HR: 3.52; 95% CI 1.14–10.84) and inversely associated with absolute lymphocyte count (HR: 0.54; 95% CI 0.33–0.87). There was no evidence of collinearity between variables included in the multivariable model (results available on Supplementary Table S3). All results are available on Table 2.

**Table 2.** Multivariable Cox proportional hazards regression analysis of baseline patients' characteristics associated with composite outcome (intubation and ICU admission or death) and mortality

	Composite outcome (intubation and ICU admission or death)			Mortality		
Characteristics	Hazard ratio^a^ (95% CIs)	*P* value	Observations	Hazard ratio^a^ (95% CIs)	*P* value	Observations
Age (years)	**2.59** (**1**.**52–4**.**40)**	**4 × 10^−4^**	327	**4.48** (**2**.**38**–**8**.**45)**	**3.52 × 10^−6^**	457
Sex Female (ref. male)	1.05 (0.53–2.12)	0.813	**Events**	1.11 (0.60–2.06)	0.739	**Events**
White blood cell count (cells/μl)	1.32 (0.97–1.80)	0.074	45	1.27 (0.90–1.78)	0.167	52
Absolute lymphocyte count (cells/μl)	**0.54 (0.33–0.87)**	**0.011**		0.63 (0.39–1.03)	0.064	
Aspartate aminotransferase (IU/l)	1.07 (0.77–1.49)	0.686		–	–	
Lactate dehydrogenase (IU/l)	**1.44 (1.04–1.98)**	**0.027**		**1.61 (1.17–2.19)**	**0.003**	
Ferritin (ng/ml)	1.18 (0.99–1.40)	0.057		**1.26** (**1**.**05–1**.**50)**	**0**.**011**	
D-dimer (μg/ml)	0.92 (0.69–1.22)	0.549		0.98 (0.74–1.28)	0.866	
C-reactive protein (mg/l)	1.23 (0.91–1.67)	0.182		0.85 (0.65–1.11)	0.228	
Il-6 (IU/ml)	1.14 (0.92–1.42)	0.226		**1.23** (**1**.**01–1**.**49)**	**0.036**	
Dyspnoea Yes (ref. No)	0.59 (0.26–1.34)	0.211		1.48 (0.80–2.75)	0.210	
Intubation Yes (ref. No)	–	–		**2.59** (**1**.**29–5**.**19)**	**0.007**	
Tocilizumab Yes (ref. No)	1.81 (0.89–3.66)	0.099		–	–	
200 mmHg < pO_2_/FiO_2_ < 300 mmHg (Ref. > 300 mmHg)	1.13 (0.40–3.20)	0.818		–	–	
100 mmHg < pO_2_/FiO_2_ < 200 mmHg (Ref. > 300 mmHg)	1.56 (0.60–4.06)	0.362		–	–	
pO_2_/FiO_2_ < 100 mmHg (Ref. > 300 mmHg)	**3.52** (**1**.**14–10**.**84)**	**0.028**		–	–	

Il-6, Interleukin-6.

^a^Continuous variables were standardised so that hazard ratios were comparable per standard deviation increase. Text in bold are the statistically significant results.

### Associations with mortality

**Fig. 2.**
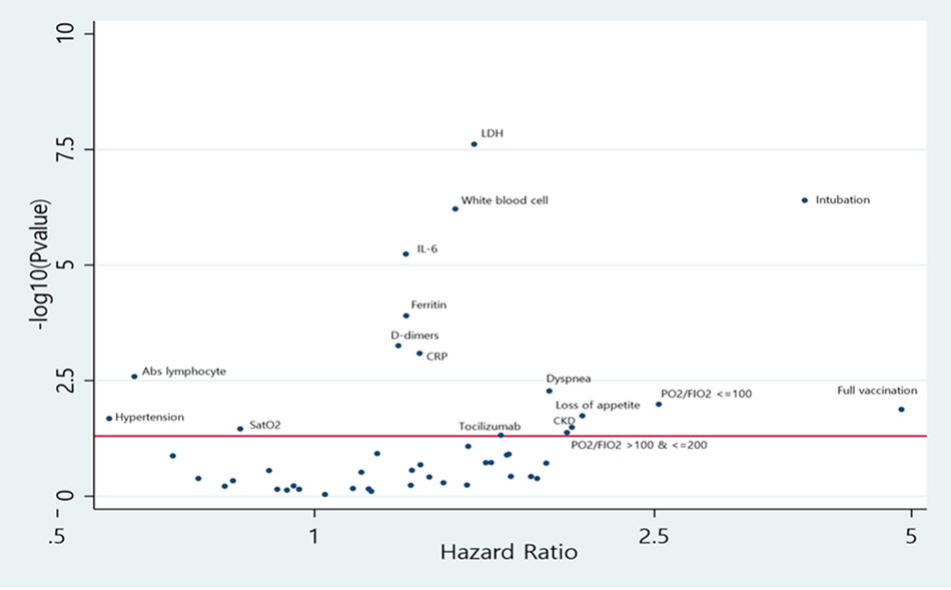
Volcano plot with Hazard ratios and −log10 (*P*-value) of each estimate from Cox regression analysis adjusted for age and sex for mortality. *The statistical threshold is 0.05 (red line in *y*-axis). The highest in *y*-axis the lowest *P*-value for the variables.

Similarly to the results for the composite outcome, the Cox regression model adjusted for age and sex revealed a statistically significant inverse association with oxygen saturation, and absolute lymphocyte count, whereas level of white blood cells count, lactate dehydrogenase, ferritin, D-Dimers, CRP, Il-6, dyspnoea, administration of tocilizumab, pO_2_/FiO_2_ ratio of 100–200 mmHg and pO_2_/FiO_2_ ratio < 100 mmHg were positively associated with death. Additionally, death was inversely associated with the presence of hypertension (HR: 0.57; 95% CI 0.36–0.92) and positively associated with CKD (HR: 2.00; 95% CI 1.06–3.78), loss of appetite (HR: 2.06; 95% CI 1.13–3.75), and intubation (HR: 3.75; 95% CI 2.25–6.25). Absolute lymphocyte count, white blood cells count, lactate dehydrogenase, ferritin, D-Dimers, CRP, IL-6, dyspnoea and intubation satisfied the FDR correction and were fitted in the multivariate model. All results are summarised on Supplementary Table S1 and presented on a volcano plot on Figure 2.

In the multivariable Cox regression model mortality was associated with age (HR: 4.48; 95% CI 2.38–8.45), levels of lactate dehydrogenase (HR: 1.61; 95% CI 1.17–2.19), ferritin (HR: 1.26; 95% CI 1.05–1.50), Il-6 (HR: 1.23; 95% CI 1.01–1.49) and intubation (HR: 2.59; 95% CI 1.29–5.19). There was no evidence of collinearity between variables included in the multivariable model (results available on Supplementary Table S4). All results are available on Supplementary Table S2.

## Discussion

This article summarises the demographic characteristics and provides insight into the clinical presentation and risk factors for intubation, ICU admission and mortality related to COVID-19 for 681 patients consecutively hospitalised due to COVID-19 in a Greek referral hospital between January 2020 and December 2021. In total, 55 patients (8%) were intubated due to respiratory failure. The overall mortality rate was 13%. In general, risk of intubation and ICU admission or death was higher for older patients, with higher lactate dehydrogenase, lower lymphocyte count and severe respiratory failure. Mortality was higher among older patients, with higher inflammatory markers and those intubated.

According to previous reports, older age, male sex, presence of comorbidities including hypertension, CVD, COPD, CKD, cancer and immunosuppression were more common in patients deceased, probably as a result of more severe disease and higher rates of respiratory failure requiring intubation and ICU admission [3, 4, 12–19, 20–23]. With regard to comorbidities, the results of this study revealed an association of CKD and immunosuppression with COVID-19-related mortality as previously reported [17, 24–26]. On the other hand, this study added a finding not commonly reported in previous reports, that the presence of hypertension was inversely associated with mortality. Nevertheless, this association did not survive multiple testing correction.

Unlike previous reports relating the presence of diabetes mellitus [16, 17], CVD [17, 27], COPD [17, 28] or cancer [17, 19] with higher risk of respiratory failure leading to intubation and higher mortality rates, no statistically significant association was established in our analysis. Similarly, despite the findings of other cohorts of COVID-19 patients which recognised obesity as a risk factor for respiratory failure leading to intubation and death [3, 4, 12, 17, 19, 24, 26, 28–30], our analysis has not found any statistically significant association. A possible explanation is that the small study sample of patients with the abovementioned conditions may reduce the ability to establish any association.

The spectrum of COVID-19 clinical presentation, including fever (83%), cough (50%), dyspnoea (27%) and gastrointestinal symptoms (32%). described in our study is similar to those in previous studies [1, 3, 4, 12, 24, 30]. Clusters of symptoms on admission were apparent with the most common symptoms' combination encompassing fever, cough and dyspnoea. Moreover, the presence of dyspnoea at admission was related with higher risk of intubation, ICU admission and death in the analysis adjusted for age and sex, as already described in previous reports [13, 19, 20, 29]. With regard to laboratory abnormalities, lymphopenia and elevated inflammatory markers were the most common laboratory findings as previously reported [12, 24, 29–31]. In addition, levels of white blood cells, lymphocytes, lactate dehydrogenase, D-Dimers, ferritin, CRP and Il-6 were associated with higher mortality rate in our analyses as previously reported [20], suggestive of a hyperinflammatory state seen in patients with the severe or critical disease [4, 13, 29, 32]. Radiographic findings were consistent with previous reports, with ground glass opacities referred in most cases [1, 12, 33]. A worth mentioning finding is that 36 patients (10%) presented with normal chest CT, a percentage comparable to the results of a previous study that reported normal chest CT in approximately 15% of individuals with early disease [12]. In the analysis adjusted for age and sex, the presence of bilateral infiltrates in CT scan was associated with higher risk of intubation or death as previously reported [34].

Interestingly, the use of corticosteroids and tocilizumab was associated with higher risk of intubation or death related to COVID-19. Both regimes are proposed by guidelines for the treatment of severe COVID-19 [10] as there is evidence of reduced mortality in severe or critically ills patients [35, 36]. The association with intubation and mortality observed in our analysis may be confounded by disease severity, as patients with more severe disease at presentation are more likely to receive treatment with corticosteroids or tocilizumab and present with poorer outcomes (intubation or death).

Another important finding of this study is that risk of intubation or death was associated with lower pO_2_/FiO_2_ ratio at presentation. This result is in accordance with previous reports that recognised the severity of hypoxaemia (lower pO_2_/FiO_2_ ratio) as a prediction factor of non-invasive ventilation failure and need for intubation as well as higher risk of death related to COVID-19 [27, 37]. Moreover, we found a stable association of intubation with COVID-19-related mortality, both in the age- and sex-adjusted and the multivariate analyses. This finding is in accordance with previous knowledge as intubation and need for ICU admission has been related with increased mortality rates [13, 24]. The association between intubation and mortality rate may be multifactorial and factors including disease severity leading to respiratory failure, patients' performance status, presence of comorbidities, extent of therapeutic regimes used prior to intubation, prolonged ICU stay and presence of complications such as secondary bacterial infections, septic shock, acute kidney injury or multiorgan failure should be taken into consideration.

This study represents an effort to systematically collect and report data on the epidemiology, clinical manifestations, outcomes and risk factors for intubation and ICU admission or mortality of all patients hospitalised with COVID-19 in a Greek referral centre for COVID-19. Nevertheless, this study should be considered in the context of its limitations. Firstly, the study involved exclusively hospitalised patients with moderate, severe and critical disease limiting the ability to capture the characteristics of non-severe disease without need of hospitalisation. Secondly, it was conducted in a single hospital centre limiting the ability to generalise the findings to other populations. Additionally, despite the rigorous effort to obtain demographic and clinical data, there is a possibility of incomplete data reporting, especially for self-reported parameters (e.g. symptoms of disease). Moreover, due to missing data for weight and height, BMI calculations were available for a smaller number of patients. In addition, given that observational data were used, our estimates may be affected by residual confounding because of differences in baseline risk and patients' characteristics that were not accounted by the measured covariates. Lastly, this report pertains exclusively to adult population.

## Conclusion

This report suggests that risk of intubation and ICU admission or death due to COVID-19 was related with older age, higher lactate dehydrogenase, lower lymphocyte count and severe respiratory failure. Mortality was higher among older patients, with higher inflammatory markers and patients intubated. Future research may focus on patients with COVID-19 at greatest risk of adverse outcomes and aim to identify medications or supportive therapies that improve their outcomes. Continuous monitoring of hospitalisation rates, clinical characteristics and outcomes of hospitalised patients would contribute to a better understanding of the clinical spectrum of COVID-19, and to guide planning and setting of priorities for the health care systems and health policies.

## Data Availability

The data that support the findings of this study are not openly available due to reasons of sensitivity and are available from the corresponding author Iro Rapti at the e-mail address: irorapti@gmail.com and telephone number: 2651099924, 6945433315 upon reasonable request.

## Supplementary material

For supplementary material accompanying this paper visit https://doi.org/10.1017/S0950268822001364. click here to view supplementary material
