# Association of patient characteristics with clinical outcomes in a cohort of hospitalised patients with SARS-CoV-2 infection in a Greek referral centre for COVID-19 – Corrigendum

**DOI:** 10.1017/S0950268823000857

**Published:** 2023-06-08

**Authors:** I. Rapti, A. Asimakopoulos, A. Liontos, M. Kosmidou, E. Christaki, D. Biros, O. Milionis, S. Tsourlos, E. Ntotsikas, E. Ntzani, E. Evangelou, K. Gartzonika, I. Georgiou, I. Tzoulaki, K. Tsilidis, H. Milionis

**Affiliations:** 1 Department of Internal Medicine, Ioannina University Hospital, University of Ioannina, Ioannina, Greece; 2 Clinical and Molecular Epidemiology Unit, Department of Hygiene and Epidemiology, School of Medicine, University of Ioannina, Ioannina, Greece; 3 Center for Evidence-Based Medicine, Department of Health Services, Policy and Practice, School of Public Health, Brown University, Providence, RI, USA; 4 Department of Epidemiology and Biostatistics, School of Public Health, Imperial College London, London, UK; 5 Microbiology Department, Faculty of Medicine, School of Health Sciences, University of Ioannina, Ioannina 45110, Greece; 6 Genetics and IVF Unit, Department of Obstetrics and Gynaecology, Medical School, University of Ioannina, Ioannina 45110, Greece

When this article was originally published in Epidemiology & Infection, the funding statement was inaccurate. This has been updated in the article to the following:

The project is co-financed by the European Regional Development Fund of the European Union and Greek national funds through 1. the Operational Program Competitiveness, Entrepreneurship and Innovation (EPAnEK), NSRF 2014-2020 (Project code MIS: OΠΣ 5047228), and 2. the Operational Programme Epirus 2014–2020 of the Prefecture of Epirus (Project code MIS: HΠ1AB-0028180).
