# Supplementary material for: Association of patient characteristics with clinical outcomes in a cohort of hospitalised patients with SARS-CoV-2 infection in a Greek referral centre for COVID-19
Source: Epidemiol Infect. 2022 Aug 16;150:e160. doi: 10.1017/S0950268822001364 (PMC9464503; doi:10.1017/S0950268822001364)
Supplement: Supplementary file 1 [file S0950268822001364sup001.docx]

**Supplement table 1.** Cox proportional hazards regression analysis adjusted for age and sex of baseline patients’ characteristics associated with the composite outcome (intubation and ICU admission or death) and mortality alone.

|  | **Composite outcome**  **(intubation and ICU admission or death)** | | **Mortality** | |
| --- | --- | --- | --- | --- |
| **Characteristics** | **Hazard ratio (95% CIs)** | ***P* value** | **Hazard ratio (95% CIs)** | ***P* value** |
| BMI (kg/m^2^) | 0.86 (0.53-1.41) | 0.55 | 0.80  (0.45-1.44) | 0.46 |
| Systolic BP (mmHg) | 1.00 (0.82-1.23) | 0.99 | 0.96  (0.77-1.19) | 0.71 |
| Diastolic BP (mmHg) | 0.93 (0.77-1.13) | 0.48 | 0.95  (0.77-1.17) | 0.60 |
| Heart rate (beats/min) | **1.23 (1.00-1.51)** | **0.05** | 1.13  (0.89-1.44) | 0.30 |
| Temperature (^o^C) | 0.97 (0.79-1.18) | 0.75 | 0.88  (0.71-1.10) | 0.28 |
| SatO_2_% | **0.81 (0.75-0.95)** | **0.01** | **0.82**  **(0.68-0.99)** | **0.03** |
| White blood cell count (cells/μL) | **1.35 (1.19-1.54)** | **4.30e-06** | **1.46**  **(1.26-1.70)** | **6.09e-07** |
| Absolute lymphocyte count (cells/μL) | **0.68 (0.52-0.90)** | **0.01** | **0.62**  **(0.45-0.84)** | **0.01** |
| Aspartate aminotransferase (IU/L) | **1.26 (1.13-1.40)** | **0.00** | 1.18  (0.96-1.46) | 0.12 |
| Lactate dehydrogenase (IU/L) | **1.48 (1.31-1.68)** | **1.24e-09** | **1.54**  **(1.32-1.79)** | **2.42e-08** |
| Ferritin (ng/mL) | **1.26 (1.13-1.39)** | **0.00** | **1.28**  **(1.15-1.42)** | **e-03** |
| D-dimer (μg/mL) | **1.24 (1.10-1.39)** | **0.00** | **1.25**  **(1.10-1.42)** | **0.01** |
| C-reactive protein (mg/L) | **1.39 (1.19-1.62)** | **0.00** | **1.33**  **(1.12-1.57)** | **0.01** |
| Il-6 (IU/ml) | **1.25 (1.14-1.36)** | **1.20e-06** | **1.28**  **(1.15-1.42)** | **5.74e-06** |
| Obesity Yes (ref. No) | 0..97 (0.60-1.57) | 0.91 | 0.90  (0.53-1.53) | 0.71 |
| Diabetes mellitus Yes (ref. No) | 1.01 (0.65-1.58) | 0.96 | 1.11  (0.68-1.81) | 0.68 |
| Hypertension Yes (ref. No) | 0.86 (0.56-1.32) | 0.50 | **0.57**  **(0.36-0.92)** | **0.02** |
| COPD Yes (ref. No) | 1.45 (0.76-2.77) | 0.25 | 1.68  (0.86-3.27) | 0.13 |
| Dyslipidemia Yes (ref. No) | 0.85 (0.57-1.27) | 0.42 | 0.93  (0.60-1.43) | 0.74 |
| Smoking Yes (ref. No) | 0.85 (0.44-1.67) | 0.64 | 0.73  (0.35-1.55) | 0.41 |
| CKD Yes (ref. No) | 1.65 (0.89-3.07) | 0.11 | **2.00**  **(1.06-3.78)** | **0.03** |
| Immunosuppression Yes (ref. No) | **1.85 (1.00-3.42)** | **0.05** | 1.69  (0.87-3.28) | 0.12 |
| Fever Yes (ref. No) | 0.91 (0.58-1.45) | 0.70 | 0.68  (0.41-1.120 | 0.13 |
| Cough Yes (ref. No) | 1.35 (0.90-2.04) | 0.15 | 1.33  (0.85-2.08) | 0.21 |
| Fatigue Yes (ref. No) | 1.42 (0.93-2.16) | 0.11 | 1.51  (0.95-2.42) | 0.08 |
| Loss of appetite Yes (ref. No) | 1.71 (0.97-3.01) | 0.06 | **2.06**  **(1.13-3.75)** | **0.02** |
| Myalgias Yes (ref. No) | 1.04 (0.58-3.15) | 0.89 | 1.36  (0.68-2.73) | 0.38 |
| Dyspnea Yes (ref. No) | **2.73 (1.83-4.06)** | **7.52e-07** | **1.88**  **(1.21-2.94)** | **0.01** |
| Anosmia Yes (ref. No) | 1.35 (0.58-3.15) | 0.49 | 1.87  (0.73-4.78) | 0.19 |
| Dysgeusia Yes (ref. No) | 1.24 (0.53-2.89) | 0.62 | 1.42  (0.50-3.99) | 0.51 |
| Nausea, diarrhea, vomiting Yes (ref. No) | 0.91 (0.57-1.45) | 0.69 | 1.03  (0.61-1.73) | 0.92 |
| Headache Yes (ref. No) | 1.75 (0.87-3.50) | 0.11 | 0.79  (0.31-1.98) | 0.61 |
| Sore throat Yes (ref. No) | 1.57 (0.72-3.41) | 0.26 | 1.30  (0.52-3.24) | 0.58 |
| Rhinorrhea Yes (ref. No) | 1.51 (0.37-6.19) | 0.57 | 1.51  (0.36-6.28) | 0.57 |
| Intubation Yes (ref. No) | - | - | **3.75**  **(2.25-6.25)** | **3.97e-07** |
| Vascular tree in bud Yes (ref. No) | 1.50 (0.76-2.94) | 0.24 | 1.16  (0.56-2.40) | 0.70 |
| Remdesivir Yes (ref. No) | 1.13 (0.74-1.74) | 0.56 | 1.30  (0.81-2.08) | 0.27 |
| Tocilizumab Yes (ref. No) | **2.91 (1.91-4.44)** | **6.47e-07** | **1.65**  **(1.00-2.72)** | 0.05 |
| Corticosteroids Yes (ref. No) | **2.11 (1.12-3.97)** | **0.02** | 1.61  (0.79-3.26) | 0.19 |
| CT chest-Unilateral infiltrate Yes (ref. No) | 1.64 (0.40-6.72) | 0.49 | 1.82  (0.43-7.65) | 0.41 |
| CT chest-Bilateral infiltrate Yes (ref. No) | **3.48 (1.05-11.55)** | **0.04** | 1.79  (0.49-6.50) | 0.38 |
| CT chest-Ground glass opacities Yes (ref. No) | 1.40 (0.48-4.06) | 0.53 | 1.17  (0.39-3.47) | 0.78 |
| Vaccination partial (ref. No) | 1.66 (0.52-5.33) | 0.39 | 1.70  (0.53-5.43) | 0.37 |
| Vaccination Full (ref. No) | 1.73 (0.53-5.65) | 0.36 | **4.86**  **(1.39-17.00)** | **0.01** |
| 200mmHg<pO_2_/FiO_2_<300mmHg (Ref.>300mmHg) | 1.77 (0.92-3.43) | 0.09 | 1.59  (0.80-3.16) | 0.19 |
| 100mmHg<pO_2_/FiO_2_<200mmHg (Ref.>300mmHg) | **2.62 (1.43-4.80)** | **0.00** | **1.97**  **(1.02-3.80)** | **0.04** |
| pO_2_/FiO_2_<100mmHg (Ref.>300mmHg) | **7.27 (3.90-13.52)** | **3.84e-10** | **2,53**  **(1.25-5.14)** | **0.01** |

BP:blood pressure, CKD:chronic kidney disease, COPD: chronic obstructive pulmonary disease, CT:computer tomography, CVD:coronary artery disease, Il-6: Interleukin-6, SatO_2_%:oxygen saturation

* Continuous variables were standardized so that hazard ratios were comparable per standard deviation increase. (6.05 kg/m^2^ for BMI, 16.90 years for age, 19.51 mmHg for systolic blood pressure, 12.69 mmHg for diastolic blood pressure, 13.69 beats/min for heart rate, 0.99^ο^C for temperature, 4.05 % for SatO_2,_ 3607.81 cells/μL for white blood cell count, 593.19 cells/μL for absolute lymphocyte count, 34.36 IU/L for aspartate aminotransferase,144.94 IU/L for lactate dehydrogenase, 673.13 ng/mL for ferritin, 2.78 μg/mL for D-dimers, 69.68 mg/L for CRP, 129.30 IU/ml for Il-6, 10.13 days for the length of hospitalization).

**Supplement table 2.** Replacement of baseline values with values of day 3.

| **Variable** | **% missing of baseline values** | **% missing after replacement with day 3 values** |
| --- | --- | --- |
| Ferritin (ng/mL) | 11,75 | 31,72 |
| D-dimer (μg/mL) | 8.96 | 18.50 |
| Il-6 (IU/ml) | 18.50 | 48.31 |
| CT chest | 29.37 | 46.40 |

**Supplement table 3.** Check for multicollinearity for composite outcome (intubation and ICU admission or death)

| **Variable** | **Variance Inflation Factor (VIF)** |
| --- | --- |
| Age (years) | 1.19 |
| Sex Female (ref. male) | 1.10 |
| White blood cell count (cells/μL) | 1.30 |
| Absolute lymphocyte count (cells/μL) | 1.15 |
| Aspartate aminotransferase (IU/L) | 1.30 |
| Lactate dehydrogenase (IU/L) | 1.73 |
| Ferritin (ng/mL) | 1.17 |
| D-dimer (μg/mL) | 1.29 |
| C-reactive protein (mg/L) | 1.31 |
| Il-6 (IU/ml) | 1.29 |
| Dyspnea Yes (ref. No) | 1.27 |
| Tocilizumab Yes (ref. No) | 1.25 |
| pO_2_/FiO_2_ | 1.62 |

**Supplement table 4.** Check for multicollinearity for mortality.

| **Variable** | **Variance Inflation Factor (VIF)** |
| --- | --- |
| Age (years) | 1.14 |
| Sex Female (ref. male) | 1.08 |
| White blood cell count (cells/μL) | 1.22 |
| Absolute lymphocyte count (cells/μL) | 1.16 |
| Lactate dehydrogenase (IU/L) | 1.40 |
| Ferritin (ng/mL) | 1.16 |
| D-dimer (μg/mL) | 1.24 |
| C-reactive protein (mg/L) | 1.31 |
| Il-6 (IU/ml) | 1.21 |
| Dyspnea Yes (ref. No) | 1.12 |
| Intubation Yes (ref. No) | 1.09 |
